# Immersion challenge of three salmonid species (family Salmonidae) with three multilocus sequence typing variants of *Flavobacterium psychrophilum* provides evidence of differential host specificity

**DOI:** 10.1111/jfd.13889

**Published:** 2023-11-16

**Authors:** Christopher Knupp, Thomas P. Loch

**Affiliations:** ^1^ Aquatic Animal Health Laboratory Michigan State University East Lansing Michigan USA; ^2^ Department of Fisheries and Wildlife, College of Agriculture and Natural Resources Michigan State University East Lansing Michigan USA; ^3^ Department of Pathobiology and Diagnostic Investigation, College of Veterinary Medicine Michigan State University East Lansing Michigan USA

**Keywords:** bacterial coldwater disease, *Flavobacterium*, host specificity, host‐pathogen interaction, rainbow trout fry syndrome, serotype

## Abstract

Bacterial coldwater disease (BCWD), caused by *Flavobacterium psychrophilum*, results in significant losses among multiple salmonid (family Salmonidae) species. Molecular epidemiology and serotyping studies have suggested that some variants are host specific; however, these associations have not been evaluated by cross‐challenging fish species with putatively host‐associated *F. psychrophilum* isolates via more natural (i.e. immersion) exposure routes. To this end, *F. psychrophilum* isolates US19‐COS, US62‐ATS and US87‐RBT, each originally recovered from diseased coho salmon (*Oncorhynchus kisutch*), Atlantic salmon (*Salmo salar*) or rainbow trout (*O. mykiss*), and belonging to a host‐associated multilocus sequence typing clonal complex (e.g. CC‐ST9, CC‐ST232 or CC‐ST10), were PCR‐serotyped, evaluated for proteolytic activity, and used to challenge adipose fin‐clipped 4‐month old Atlantic salmon, coho salmon and rainbow trout via immersion. Findings showed US87‐RBT caused disease and mortality only in rainbow trout (e.g. 56.7% survival probability). US19‐COS and US62‐ATS caused more mortality in coho salmon and Atlantic salmon but also caused disease in both other host species, albeit to a lesser extent. Observed survival differences may be due to variant antigenic/virulence determinants as differences in serotype and proteolytic activity were discovered. Collectively, results highlight the intricacies of *F*. *psychrophilum*‐host interactions and provide further in vivo evidence that some *F. psychrophilum* MLST variants are host specific, which may have implications for the development of BCWD prevention and control strategies.

## 1 INTRODUCTION

*Flavobacterium psychrophilum*, causative agent of bacterial coldwater disease (BCWD) and rainbow trout fry syndrome (RTFS), causes substantial mortality and economic losses in farm and hatchery‐reared salmonids (family Salmonidae) worldwide (Loch & Faisal, 2017). Rainbow trout (*Oncorhynchus mykiss*) and coho salmon (*O. kisutch*) are considered most susceptible (Holt, 1987), particularly at early life stages when mortality is highest (e.g. 50–90%; Barnes & Brown, 2011). Likewise, BCWD epizootics in farmed Atlantic salmon (*Salmo salar*) are also common (Avendano‐Herrera et al., 2020; Macchia et al., 2022; Nilsen et al., 2011).

Multilocus sequence typing (MLST) has become a widespread tool for molecular epidemiological studies on *F. psychrophilum* (Avendaño‐Herrera et al., 2014; Fujiwara‐Nagata et al., 2013; Li et al., 2021; Nicolas et al., 2008; Nilsen et al., 2014). To date, >1500 isolates recovered in 18 countries on five continents have been genotyped via MLST, revealing the existence of >260 distinct sequence types (STs; https://pubmlst.org/fpsychrophilum). A common finding among MLST‐based epidemiological studies is that some *F. psychrophilum* MLST clonal complexes (CCs) are most associated with a single host species (Avendaño‐Herrera et al., 2014; Fujiwara‐Nagata et al., 2013; Knupp et al., 2019; Li et al., 2021; Nilsen et al., 2014; Sebastiao et al., 2020; Van Vliet et al., 2016). For example, most *F. psychrophilum* isolates belonging to CC‐ST10, which is the largest and most reported CC worldwide, are recovered from rainbow trout (Avendaño‐Herrera et al., 2014; Knupp et al., 2019; Li et al., 2021; Nilsen et al., 2014; Van Vliet et al., 2016). Similarly, most *F. psychrophilum* isolates belonging to CC‐ST9 are recovered from coho salmon, whereas all isolates in CC‐ST232 have been recovered from Atlantic salmon (Avendaño‐Herrera et al., 2014; Fujiwara‐Nagata et al., 2013; Knupp et al., 2019; Nilsen et al., 2014; Van Vliet et al., 2016).

In addition to being genetically diverse, *F. psychrophilum* also exhibits serotypic diversity (Holt, 1987; Lorenzen & Olesen, 1997; Pacha, 1968). Although classical serotyping methods remain in use, a multiplex PCR‐based serotyping assay was recently developed by Rochat et al. (2017), which detects four molecular serotypes (e.g. Type‐0–Type‐3) and that was later amended by Avendano‐Herrera et al. (2020) to detect a fifth molecular serotype (e.g. Type‐4). This molecular serotyping assay has been applied to >350 *F. psychrophilum* isolates, and findings suggest some serotypes have host associations. For instance, *F. psychrophilum* isolates recovered from rainbow trout most commonly belong to Type‐1 and Type‐2, whereas isolates recovered from Atlantic salmon and coho salmon are most frequently associated with Type‐2 and Type‐4, or Type‐0, respectively (Avendano‐Herrera et al., 2020; Calvez et al., 2021; Knupp, Kiupel, et al., 2021; Li et al., 2021; Rochat et al., 2017; Sundell et al., 2019). Likewise, *F. psychrophilum* isolates recovered from ayu (*Plecoglossus altivelis*) have been exclusively reported as Type‐3 (Rochat et al., 2017).

The putative host associations of some *F. psychrophilum* geno‐ and sero‐variants are largely based on observational associations in naturally infected fish. However, several studies have directly or indirectly investigated such associations under in vivo laboratory conditions (Ekman & Norrgren, 2003; Fredriksen et al., 2016; Holt, 1987), though most have used a less natural exposure route (e.g. injection) that bypasses important immune defences (Dash et al., 2018; Fast et al., 2002). In contrast, at least one study reported the virulence of two host‐associated *F. psychrophilum* variants (e.g. US19‐coho salmon in ST9 and US53‐rainbow trout in ST78) in coho salmon following laboratory immersion exposure (Knupp, Kiupel, et al., 2021). This study revealed that only the coho salmon‐recovered isolate caused disease and mortality in coho salmon, whereas the rainbow trout‐recovered isolate did not, despite proving virulent to rainbow trout in a previous study (Knupp, Faisal, et al., 2021). However, a study has yet to simultaneously cross‐challenge multiple salmonid species of a similar age with multiple putatively host specific *F. psychrophilum* variants, thereby leaving a gap of knowledge in BCWD ecology with potentially important implications for future prevention and control strategies. To further investigate *F. psychrophilum* host specificity, in vitro and in vivo experiments were designed to elucidate the interactions between *F. psychrophilum* and three salmonid species.

## 2 MATERIALS AND METHODS

### 2.1 *Flavobacterium psychrophilum* isolates

Three *F. psychrophilum* isolates (e.g. US19, US62 and US87) belonging to three MLST STs (e.g. ST13, ST277 and ST275) within three MLST CCs (e.g. CC‐ST9, CC‐ST232 and CC‐ST10) that are geographically widespread (i.e. detected on 2–4 continents; Avendaño‐Herrera et al., 2014; Fujiwara‐Nagata et al., 2013; Knupp et al., 2019; Li et al., 2021; Nilsen et al., 2014) and exclusively or nearly exclusively recovered from one of three economically important salmonid species (e.g. coho salmon, rainbow trout or Atlantic salmon) were selected from this study. In addition, recent studies have demonstrated US19 is virulent to coho salmon via immersion (Knupp, Kiupel, et al., 2021), and US87 to rainbow trout via injection (Knupp, Faisal, et al., 2021).

### 2.2 Molecular serotyping

Like MLST ST, molecular serotype may also be indicative of some *F. psychrophilum* host associations (Avendano‐Herrera et al., 2020; Rochat et al., 2017; Sundell et al., 2019); therefore, the molecular serotypes of *F. psychrophilum* isolates US62‐ATS and US87‐RBT were determined using an adapted version of the mPCR‐based serotyping approach as described by Knupp, Kiupel, et al. (2021). In brief, each 50‐μL mPCR reaction contained 25 μL of 2× GoTaq® Green Master Mix (Promega), 20 ng of DNA template, 0.1 μM of each control primer and 0.5 μM of each primer targeting the molecular serotypes, with the remaining volume consisting of nuclease‐free water. Sterile nuclease‐free water functioned as a negative control, while *F. psychrophilum* isolates US19‐COS, FP900406, CSF259‐93, US104 and US515 acted as positive controls for Type‐0, Type‐1, Type‐2, Type‐3 and Type‐4 respectively (Knupp, Kiupel, et al., 2021; Knupp, Faisal, et al., 2021; Loch and Knupp, unpublished). The mPCR cycling conditions outlined by Rochat et al. (2017) were employed using an Eppendorf Mastercycler pro thermal cycler. A 1.5% agarose gel containing 1X SYBR Safe DNA gel stain was used to separate 5 μL of the amplified PCR product via electrophoresis for 35 min at 100 V. A 1‐Kb Plus DNA Ladder (ThermoFisher Scientific) served as the molecular size standard. The gel was examined under UV transillumination to estimate amplicon size and assign mPCR serotypes (e.g. Type‐0, 188 bp; Type‐1, 188 and 549 bp; Type‐2, 188 and 841 bp; Type‐3, 188 and 361 bp; and Type‐4, 188 and 992 bp; Avendano‐Herrera et al., 2020; Rochat et al., 2017).

### 2.3 Characterization of proteolytic activity

*Flavobacterium psychrophilum* can proteolyze multiple components (e.g. casein, gelatin, elastin and collagen) representative of host connective and muscle tissue, and therefore proteolytic activity has been suggested as a virulence determinant (Knupp, Kiupel, et al., 2021; Madsen & Dalsgaard, 1998; Nakayama et al., 2016; Rochat et al., 2019). Moreover, proteolysis of some components (e.g. elastin and collagen) may be more commonly associated with *F. psychrophilum* isolates with different host associations (e.g. Knupp, Kiupel, et al., 2021; Nakayama et al., 2016; Rochat et al., 2019). Therefore, the proteolytic activities of US62‐ATS and US87‐RBT were assessed on tryptone yeast extract salt medium (TYES; Holt, 1987) supplemented with casein, elastin or gelatin as described previously (Knupp, Kiupel, et al., 2021; Sundell et al., 2019). The proteolytic activity of US19‐COS has been previously assessed on these substrates (Knupp, Faisal, et al., 2021; Knupp, Kiupel, et al., 2021) but was included for comparison purposes. Additionally, collagenase activity was assessed by supplementing TYES with 5% (w/v) collagen from bovine Achilles tendon (ThermoFisher). Briefly, *F. psychrophilum* was revived from cryostock (maintained at −80°C) on TYES, which was modified according to Michel et al. (1999), incubated for 48 h at 15°C and then visually inspected for purity. Each *F. psychrophilum* isolate was inoculated into 80 mL of analogous broth and incubated with constant shaking (180 rpm) for 48 h at 15°C. Bacteria were harvested via centrifugation (2571 × ***g***, 15 min) and adjusted to an optical density at 600 nm (OD_600_) corresponding to 1 × 10^9^ cfu/mL using a spectrophotometer (WPA, Inc.) and sterile 0.65% saline. To quantify *F. psychrophilum* concentrations, serial dilutions in 10‐fold increments (up to 100,000,000‐fold) were plated on modified TYES in duplicate, and then incubated for 7 days, after which final colony counts were performed. To determine proteolytic activity, 10 μL of each *F. psychrophilum* isolate was spotted in triplicate on the surface of the four media, allowed to dry and then incubated for 7 days at 15°C. The colony and clear zone diameters were summed and then divided by the colony diameter to yield the clear zone ratio (CZR; Sundell et al., 2019).

### 2.4 In vivo virulence assessment of *Flavobacterium psychrophilum* isolates US19‐COS, US62‐ATS and US87‐RBT to Atlantic salmon, coho salmon and rainbow trout

#### 2.4.1 Origin of fish for challenge experiments

Embryonated Atlantic salmon and rainbow trout eggs were sourced from a commercial egg distributor, while embryonated coho salmon eggs were procured from Platte River State Fish Hatchery. For all three fish species, the number of contributing parents are unknown. Coordination occurred so that all eggs from the three species arrived at the Michigan State University—University Research Containment Facility on the same day. In brief and upon receipt, eggs were disinfected with 100 ppm iodophor solution (pH 7.30) for 10 min before being placed in a vertical incubator supplied with UV‐treated, sand‐filtered well water maintained at 12 ± 1°C until hatching. Sac‐fry were then moved to aerated flow‐through tanks (40 L; 12 ± 1°C) and, once exogenous feeding commenced, were given a continuous supply of appropriately sized commercial trout food (Skretting) via an automatic feeder. After 8 weeks, fish were hand‐fed twice daily and the water volume in the tanks was increased (400 L; 12 ± 1°C). Tanks were cleaned and siphoned daily to remove waste and any uneaten food. Before the challenge experiment, a sample of fish from each species were cultured to screen for bacterial infections (Knupp, Kiupel, et al., 2021), including those caused by *F. psychrophilum*, and confirmed to be bacterial infection‐free.

#### 2.4.2 *Flavobacterium psychrophilum* inoculum preparation for immersion challenge

*Flavobacterium psychrophilum* isolates US19‐COS, US62‐ATS and US87‐RBT were revived from cryostock (maintained at −80°C) on *F. psychrophilum* medium‐A (FPM‐A; Knupp, 2023), incubated for 48 h at 15°C and then visually inspected for purity. Each isolate was inoculated into 3 L of FPM‐A broth, incubated, harvested and adjusted to 10^9^ cfu/mL using sterile 0.65% saline as described in section 2.3. *F. psychrophilum* concentrations were verified via plate counts.

#### 2.4.3 Immersion challenge experiment

The ability of *F. psychrophilum* isolates US19‐COS, US62‐ATS and US87‐RBT to infect and cause disease in 4‐month‐old Atlantic salmon (mean weight 1.1 g), coho salmon (mean weight 5.0 g) and rainbow trout (mean weight 7.8 g) was assessed via immersion exposure. Three‐hundred and sixty total fish, including 120 Atlantic salmon, 120 coho salmon and 120 rainbow trout were anaesthetised in sodium bicarbonate‐buffered (200 mg/L) tricaine methanesulfonate (MS‐222; Syndel) at a concentration of 100 mg/L, adipose fin‐clipped using sharp sterile scissors (Holt, 1987) and then allowed to recover in aerated water. Fish (*n* = 15, in duplicate) of each species (*n* = 3) were immersed for 30 min in aerated water (12 ± 1°C) containing 10^7^ cfu/mL of US19‐COS, US62‐ATS or US87‐RBT. Control fish (*n* = 15, in duplicate) of each species (*n* = 3) were immersed in an identical volume of water only. After bacterial exposure, fish were net (fine mesh) transferred into aerated flow‐through glass aquaria (37.8 L; *n* = 15 fish per aquarium, in duplicate) supplied with ultraviolet light‐treated, sand‐filtered well water (12 ± 1°C; 1.5 L/min).

Fish were monitored daily for 25 days and cared for as described in section 2.4.1; mortalities were necropsied and clinically examined, and multiple tissues (e.g. external ulcers and kidney) were bacteriologically analysed for *F. psychrophilum* on FPM‐A. Surviving fish (e.g. 25 days post‐exposure) were sacrificed via MS‐222 overdose (250 mg/L) and analysed similarly. All challenge experiments were conducted in accordance with the MSU‐Institutional Animal Care and Use Committee (AUF: 201900312).

Representative isolates recovered from dead and surviving fish were confirmed as *F. psychrophilum* via endpoint PCR (Toyama et al., 1994; Van Vliet et al., 2015). Likewise, *F. psychrophilum* MLST STs of representative isolates were confirmed via PCR amplification and Sanger‐sequencing of all seven *F. psychrophilum*‐specific MLST loci as previously described (Knupp et al., 2019).

### 2.5 Data analysis

The Kruskal–Wallis test was used to examine median CZR differences among isolates (e.g. US19‐COS, US62‐ATS and US87‐RBT) for the tested media (e.g. caseinase, collagenase, elastase and gelatinase). If the null hypothesis of no difference in median CZR among isolates was rejected, pairwise comparisons of median CZR between isolates were carried out using Dunn's test and applying the Bonferroni correction for multiple comparisons (*α* = 0.05). The Kruskal–Wallis tests and Dunn's tests were conducted using PROC npar1way and custom SAS code, respectively.

Kaplan–Meier plots (Kaplan & Meier, 1958) with 95% confidence intervals were generated using PROC LIFETEST and SGPLOT to visualize Atlantic salmon, coho salmon and rainbow trout survival probabilities over time after exposure to either US19‐COS, US62‐ATS or US87‐RBT.

Relative risk of death among fish species exposed to each isolate was assessed using Cox proportional hazards regression models. Fish species was treated as a categorical variable. Comparisons of hazard ratios (i.e. risk of death) between fish species were evaluated for each isolate. If mortality did not occur among experimental units (i.e. aquaria) for one or more fish species, the Cox proportional hazards regression model was replaced by pairwise comparisons of survival rate between fish species on day 25 (i.e. the end of the experiment) using two‐sample *z*‐tests. The Cox proportional hazards regression models and pairwise comparisons were conducted using PROC PHREG and custom code. All statistical analyses were performed using SAS® Version 9.4; (*α* = 0.05).

## 3 RESULTS

### 3.1 Molecular serotype

The three *F. psychrophilum* isolates each belonged to a different molecular serotype, whereby US19‐COS, US62‐ATS and US87‐RBT were identified as Type‐0 (Knupp, Kiupel, et al., 2021), Type‐1 and Type‐2, respectively.

### 3.2 Proteolytic activity

*Flavobacterium psychrophilum* isolates US19‐COS, US62‐ATS and US87‐RBT all proteolyzed casein, collagen and gelatin; however, only US87‐RBT proteolyzed elastin (Table 1). The Kruskal–Wallis test indicated that there were overall significant differences among the isolates in median CZR for elastase (χ^2^ = 8.00; df = 2; *p*‐value = .0183) and gelatinase (χ^2^ = 7.71; df = 2; *p*‐value = .0211). However, the null hypothesis of no difference in median CZR among the isolates for caseinase (χ^2^ = 5.84; df = 2; *p*‐value = .0538) and collagenase (χ^2^ = 5.73; df = 2; *p*‐value = .0571) could not be rejected (i.e. no significant difference in caseinase and collagenase activity among the isolates). For elastase, the median CZR produced by US87‐RBT was significantly greater than US19‐COS (*Z*‐value = 3.4641; *p*‐value = .0008) and US62‐ATS (*Z*‐value = 3.4641; *p*‐value = .0008; Table 1). For gelatinase, the median CZR produced by US19‐COS and US87‐RBT were both significantly greater than US62‐ATS (US19‐COS vs. US62‐ATS: *Z*‐value = 2.3094, *p‐*value = .0314; US87‐RBT vs. US62‐ATS: *Z*‐value = 3.4641, *p*‐value = .0008; Table 1).

**TABLE 1:** *Flavobacterium psychrophilum* isolates used in this study for in vivo challenge experiments against Atlantic salmon (*Salmo salar*), coho salmon (*Oncorhynchus kisutch*) and rainbow trout (*O. mykiss*) and for the assessment of proteolytic activity, which is presented as a ratio of the median clear zone diameter to the colony diameter (in mm) ± SE.

Isolate ID	Host of origin	ST	CC	Caseinase	Protease clear zone ratio ± SE		
					Collagenase	Gelatinase	Elastinase
US19	Coho salmon	ST13	CC‐ST9	3.00 (0.13)*	2.00 (0.13)*	2.31 (0.05)*	1.00 (0.00)*
US62	Atlantic salmon	ST277	CC‐ST232	3.88 (0.30)*	2.00 (0.12)*	1.79 (0.00)**	1.00 (0.00)*
US87	Rainbow trout	ST275	CC‐ST10	3.44 (0.00)*	3.44 (0.00)*	3.50 (0.00)***	2.00 (0.00)**

*Note*: Median clear zone ratios for a particular enzyme (e.g. caseinase, gelatinase or elastinase) containing an identical symbol (e.g. *, **, ***) are not significantly different (*α* = 0.05) and a ratio of 1.00 ± 0.00 indicates no protease activity.

*Abbreviations*: SS, sequence type; CC, clonal complex.

### 3.3 Virulence of *Flavobacterium psychrophilum* isolates US19‐COS, US62‐ATS and US87‐RBT to Atlantic salmon, coho salmon and rainbow trout

#### 3.3.1 Negative control Atlantic salmon, coho salmon and rainbow trout

Throughout the course of the in vivo challenge experiments, no negative control fish, in any of the three fish species, died.

#### 3.3.2 *Flavobacterium psychrophilum* isolate US87‐RBT

**FIGURE 1:**
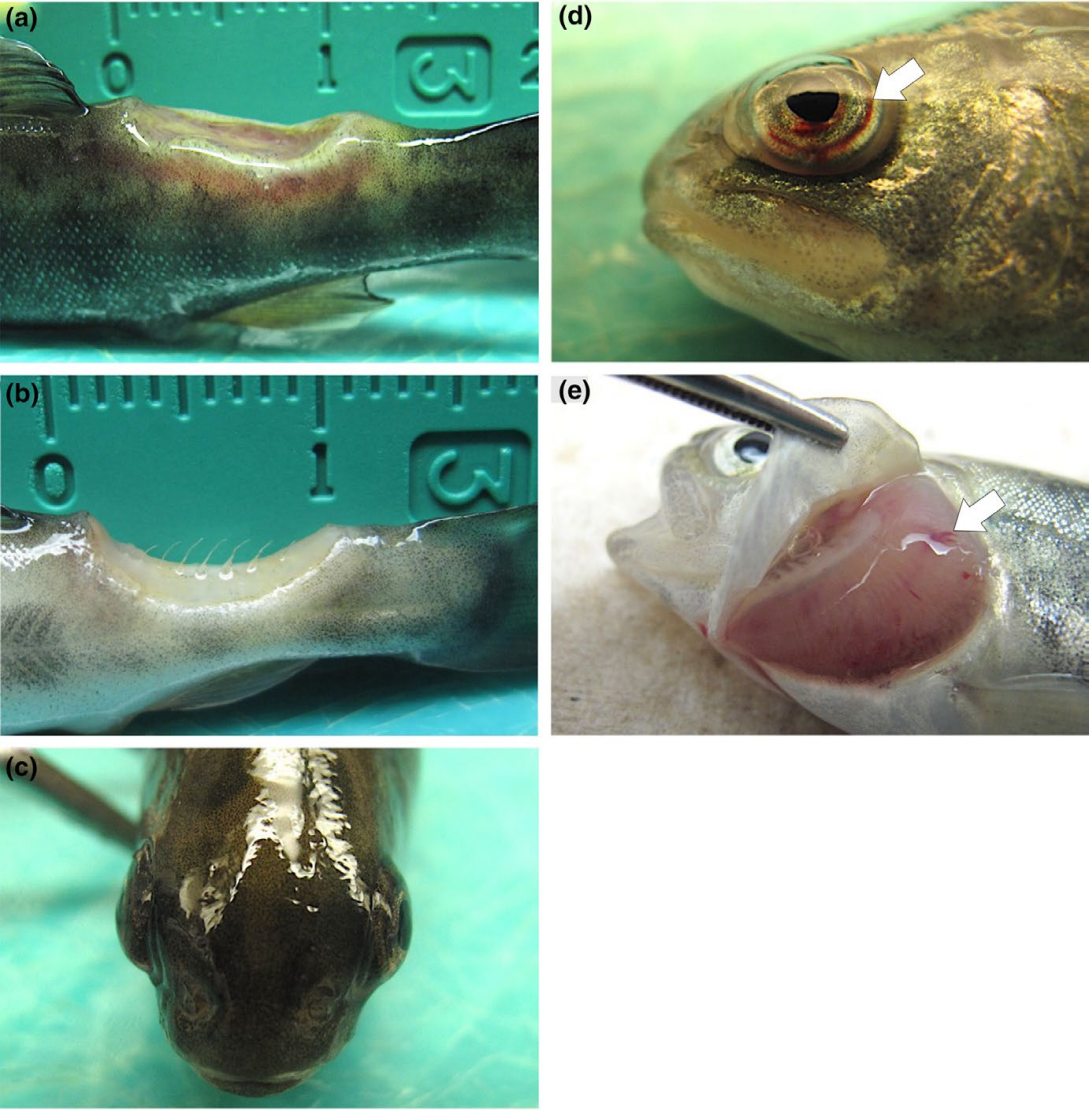
Gross external lesions in rainbow trout (*Oncorhynchus mykiss*) following immersion challenge with *Flavobacterium psychrophilum* isolate US87‐RBT. (a) Focally extensive ulceration of the caudal peduncle that penetrated the underlying musculature. (b) Focally extensive ulceration of the caudal peduncle that penetrated the underlying musculature and exposed the vertebral column. (c) Bilateral exophthalmia. (d) Unilateral exophthalmia with intraocular ecchymosis (arrow). (e) Gill pallor with ecchymoses and petechiae (arrow).

**FIGURE 2:**
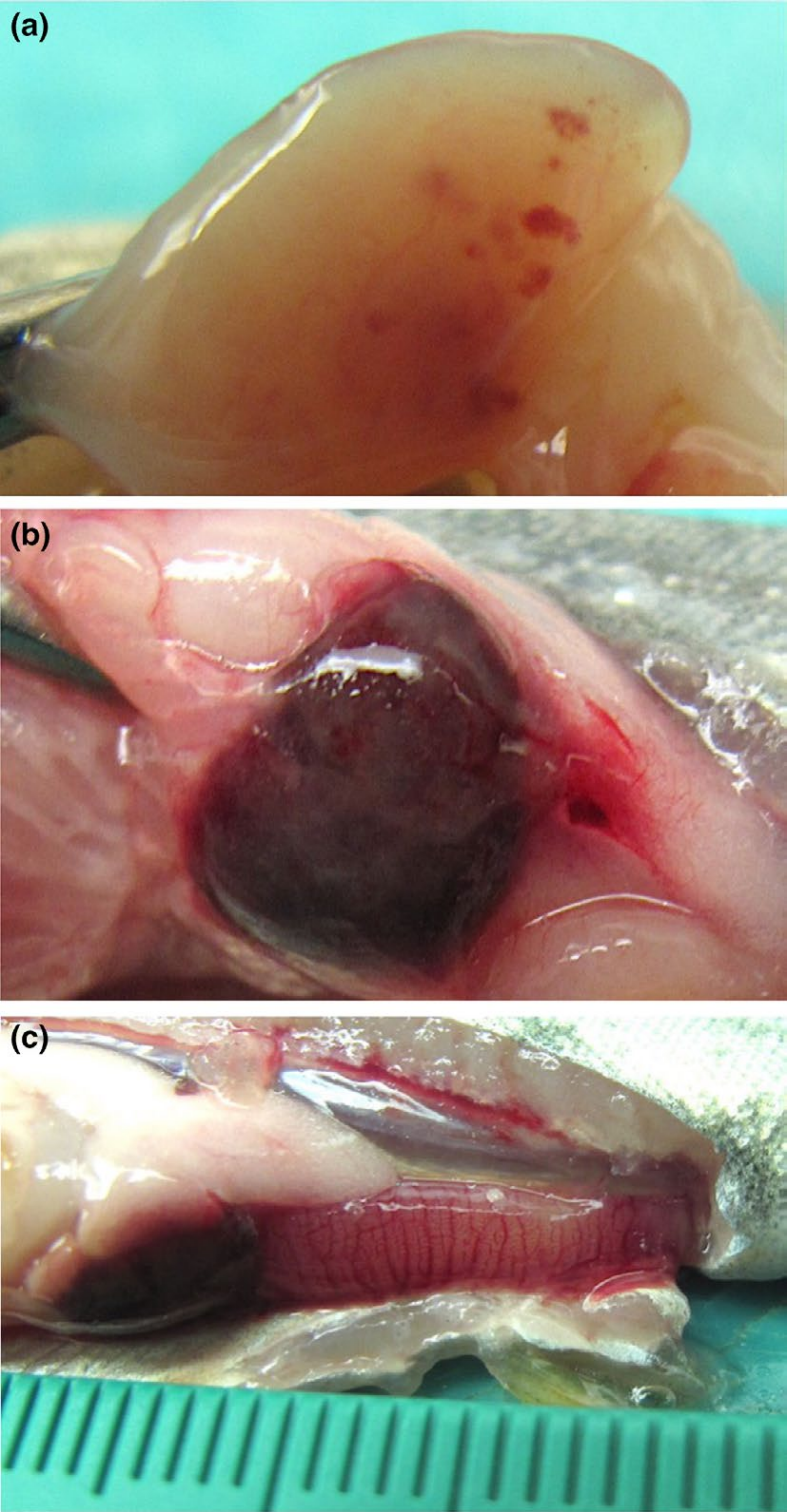
Gross internal lesions in rainbow trout (*Oncorhynchus mykiss*) following immersion challenge with *Flavobacterium psychrophilum* isolate US87‐RBT. (a) Pale liver with multifocal ecchymoses. (b) Severe splenic swelling with perisplenic haemorrhage. (c) Severe intestinal haemorrhage with accompanying peri‐intestinal haemorrhage.

Following immersion exposure to *F. psychrophilum* isolate US87‐RBT, rainbow trout was the only fish species to develop gross signs of BCWD. Disease signs were evident as early as 9 days post‐exposure in the form of focally extensive dermal ulceration of the caudal peduncle that penetrated the underlying musculature (Figure 1a). As disease progressed, the caudal peduncle ulceration deepened further into the underlying musculature, exposing the vertebral column (Figure 1b). Rainbow trout also exhibited uni‐ or bilateral exophthalmia with or without intraocular ecchymosis (Figure 1c,d; Table 2) and/or gill pallor with or without ecchymosis and/or petechiae (Figure 1e). Internally, rainbow trout infected with US87‐RBT presented with visceral organ (e.g. heart, liver and kidney) pallor, multifocal hepatic ecchymoses (Figure 2a), severe splenic swelling with perisplenic haemorrhage (Figure 2b), and severe intestinal haemorrhage with accompanying peri‐intestinal haemorrhage (Figure 2c; Table 2). In a subset of surviving rainbow trout (i.e. sacrificed 25 days post‐exposure), unilateral exophthalmia and caudal peduncle ulceration was present. Internally, most surviving rainbow trout had moderate to severe splenic swelling. Surviving Atlantic salmon and coho salmon remained apparently healthy.

**TABLE 2:** Proportion of dead Atlantic salmon (*Salmo salar*), coho salmon (*Oncorhynchus kisutch*) and rainbow trout (*O. mykiss*) with a range of gross external and internal bacterial coldwater disease signs following exposure to *Flavobacterium psychrophilum* isolates US19‐COS, US62‐ATS and US87‐RBT.

Isolate	Host species^a^	External disease signs		Internal disease signs		
		Haemorrhage surrounding caudal peduncle ulcer	Exophthalmia	Visceral organ haemorrhage	Splenic swelling	Intestinal haemorrhage
US19‐COS	Atlantic salmon	0/15	0/15	1/15	0/15	0/15
	Coho salmon	0/28	0/28	2/28	25/28	3/28
	Rainbow trout	7/14	0/14	1/14	14/14	3/14
US62‐ATS	Atlantic salmon	0/29	0/29	0/29	27/29	0/29
	Coho salmon	0/10	0/10	0/10	10/10	0/10
	Rainbow trout	12/26	0/26	3/26	26/26	0/26
US87‐RBT	Atlantic salmon	–	–	–	–	–
	Coho salmon	–	–	–	–	–
	Rainbow trout	0/13	3/13	6/13	13/13	3/13

^a^ All dead fish with caudal peduncle ulceration, gill pallor and visceral organ pallor.

**FIGURE 3:**
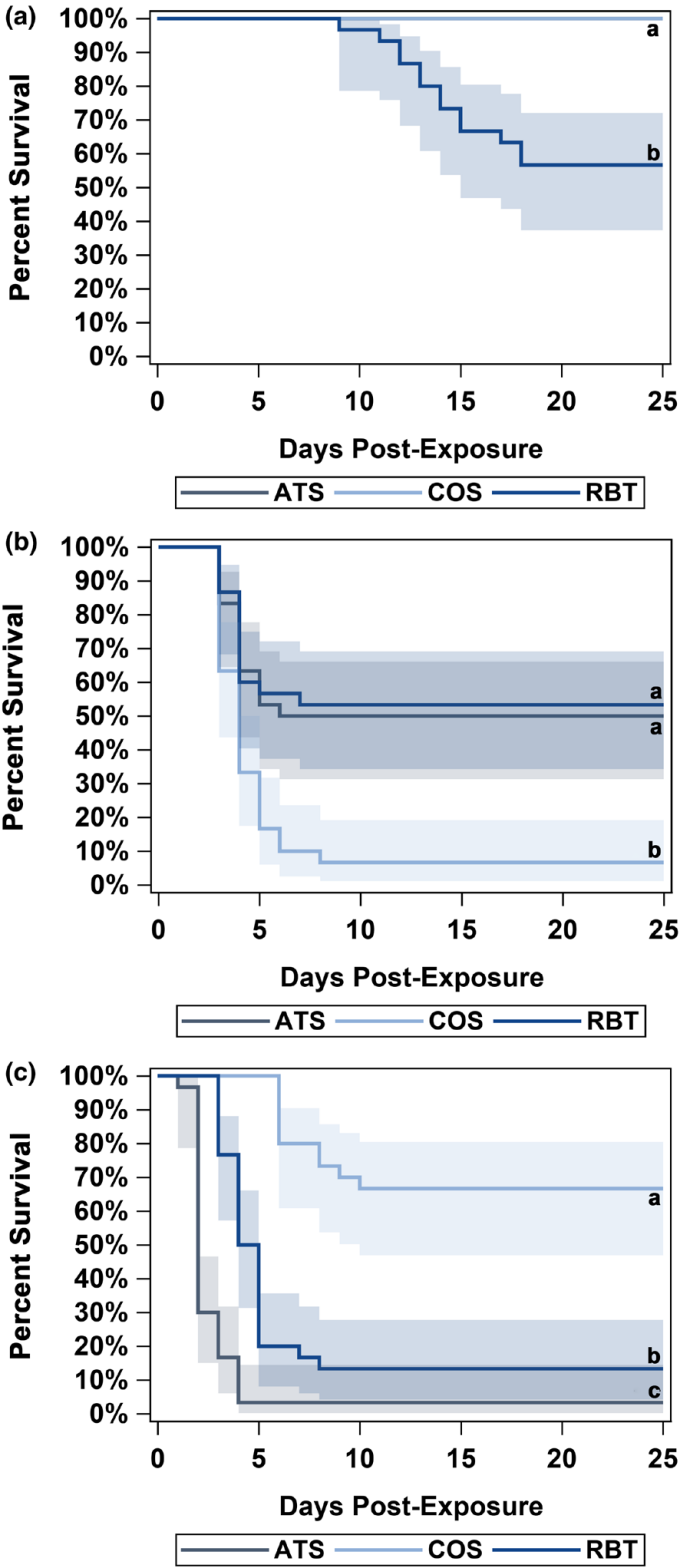
Kaplan–Meier survival curves of Atlantic salmon (*Salmo salar*; ATS), coho salmon (*Oncorhynchus kisutch*; COS) and rainbow trout (*O. mykiss*; RBT) over a 25‐day period following immersion challenge with *Flavobacterium psychrophilum* isolates (a) US87‐RBT, (b) US19‐COS and (c) US62‐ATS. Shaded regions depict 95% confidence intervals. Lines with different letters (e.g. a, b, c) indicate significant differences in survival (*α* = 0.05).

Overall survival was 56.7% in rainbow trout and 100% in Atlantic salmon and coho salmon (i.e. no Atlantic salmon or coho salmon died; Figure 3a). Rainbow trout mortality began 9 days post‐exposure and peaked on day 18 (Figure 3a). Because Atlantic salmon and coho salmon experienced no mortality, the Cox proportional hazards regression model could not be used to compare risk of death. Rainbow trout survival was significantly lower than both Atlantic salmon and coho salmon (*Z*‐scores = 4.7897; *p*‐values < .0001).

#### 3.3.3 *Flavobacterium psychrophilum* isolate US19‐COS

**FIGURE 4:**
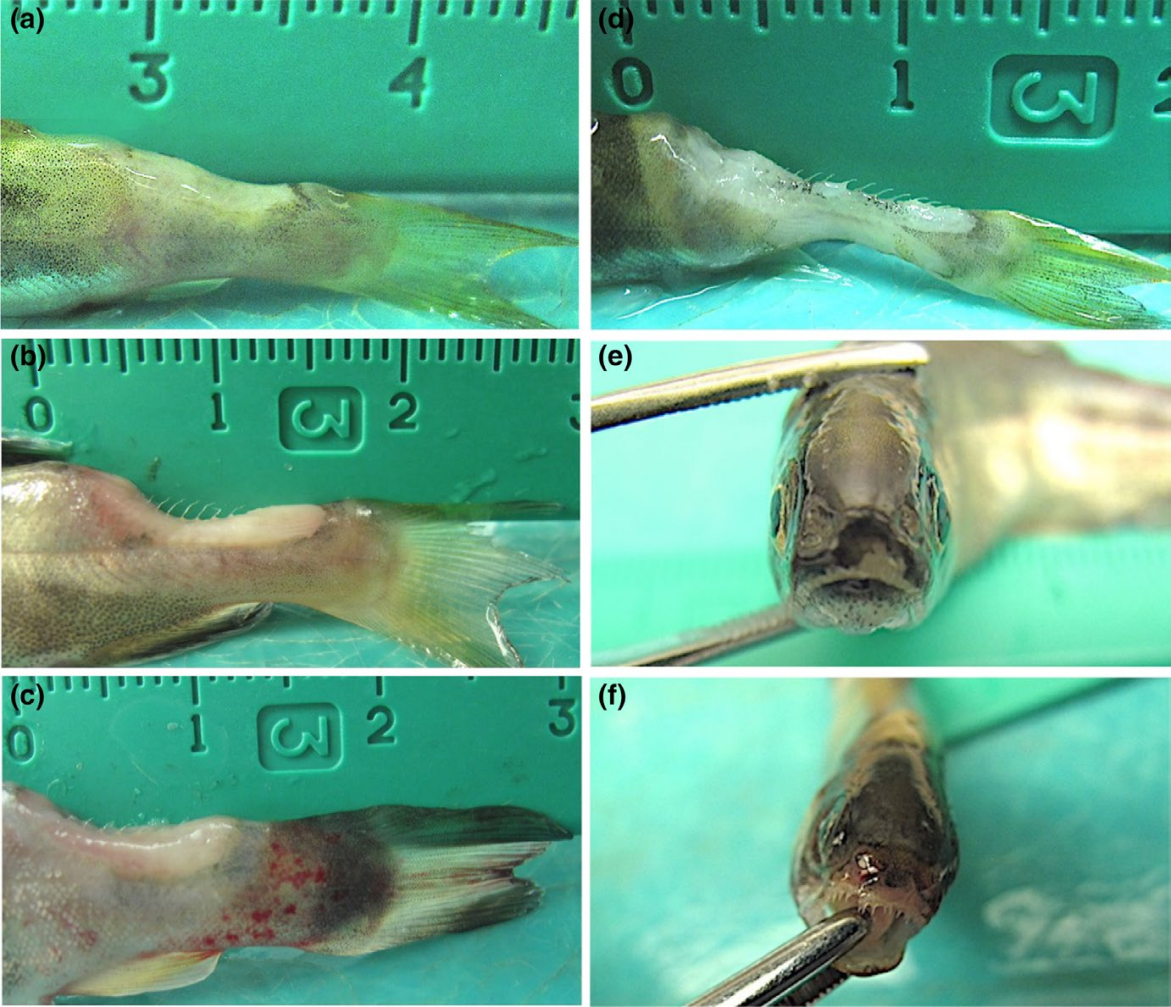
Gross external lesions in Atlantic salmon (*Salmo salar*), coho salmon (*Oncorhynchus kisutch*) and rainbow trout (*O. mykiss*) following immersion challenge with *Flavobacterium psychrophilum* isolate US19‐COS. (a) Atlantic salmon with shallow focally extensive dermal ulceration of the caudal peduncle. (b) Coho salmon with focally extensive dermal ulceration of the caudal peduncle that penetrated the underlying musculature. (c) Rainbow trout with focally extensive dermal ulceration of the caudal peduncle that penetrated the underlying musculature. Diffuse ecchymoses and petechiae surround the ulcer. (d) Atlantic salmon with focally extensive dermal ulceration of the caudal peduncle, exposing the vertebral column. (e) and (f) Peri‐oral ulceration in coho salmon (e) and rainbow trout (f).

**FIGURE 5:**
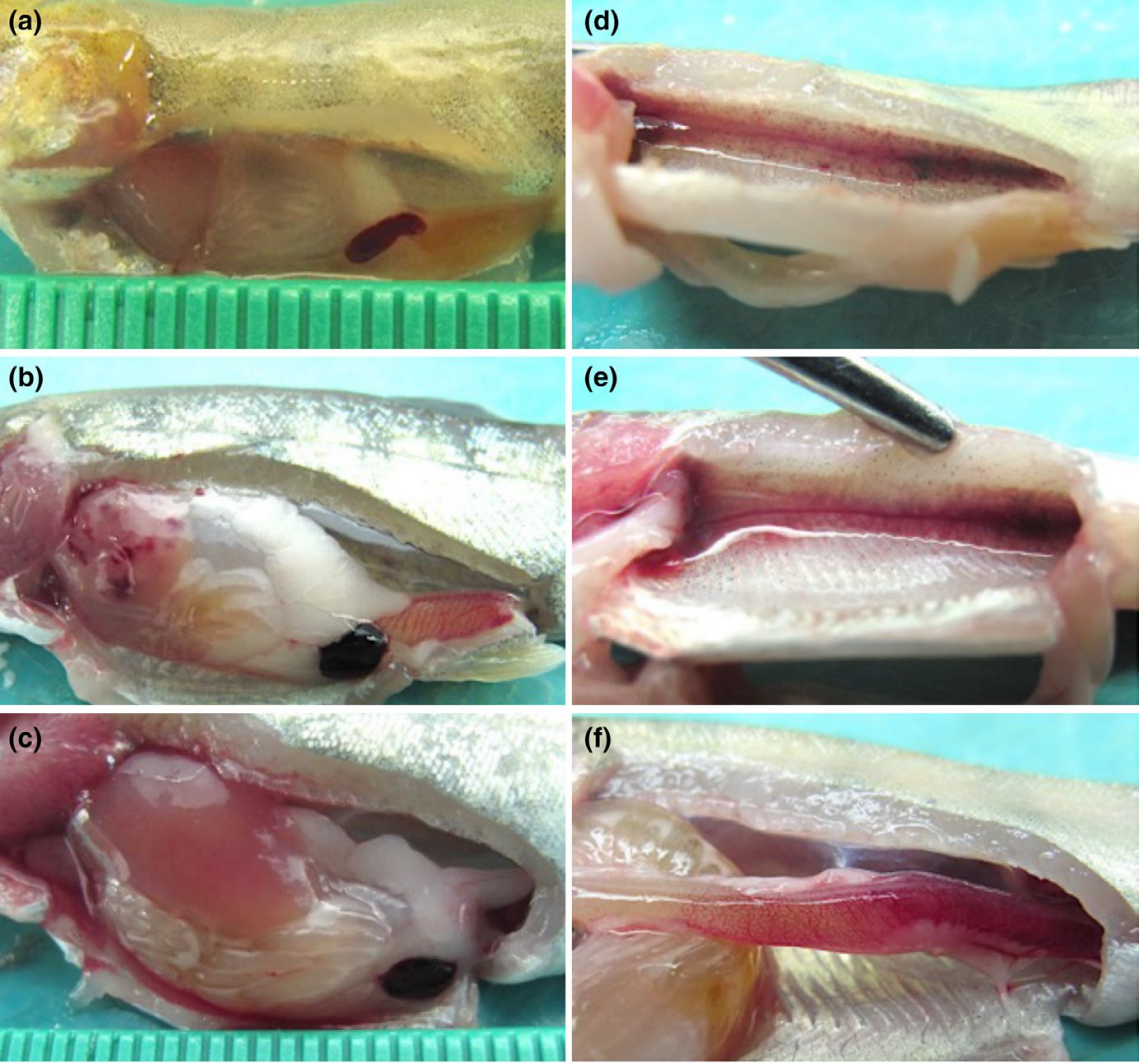
Gross internal lesions in Atlantic salmon (*Salmo salar*), coho salmon (*Oncorhynchus kisutch*) and rainbow trout (*O. mykiss*) following immersion challenge with *Flavobacterium psychrophilum* isolate US19‐COS. (a) Atlantic salmon with liver pallor. (b) Coho salmon with pale liver and multifocal ecchymoses, splenic swelling and intestinal haemorrhage. (c) Rainbow trout with liver pallor and splenic swelling. (d) Coho salmon with renal pallor. (e) Rainbow trout with renal pallor and diffuse ecchymoses. (f) Rainbow trout with intestinal haemorrhage.

Atlantic salmon, coho salmon and rainbow trout exposed to US19‐COS (ST13, CC‐ST9) developed similar gross signs of BCWD as early as 4 days post‐exposure in the form of focally extensive dermal ulceration of the caudal peduncle that penetrated into the underlying muscle; however, and early in the disease course, ulcerations were shallower (i.e. not exposing the vertebral column) in Atlantic salmon compared to coho salmon and rainbow trout (Figure 4a–c). In rainbow trout only, the tissues surrounding the ulcer frequently had diffuse ecchymoses and petechiae (Figure 4c; Table 2). As disease progressed, caudal peduncle ulcerations deepened in all species, including Atlantic salmon (Figure 4d). Another external BCWD sign common to all species was gill pallor with or without ecchymoses and petechiae. In contrast, peri‐oral ulceration was apparent in coho salmon and rainbow trout only (Figure 4e,f). Internally, most gross disease signs caused by US19‐COS were similar among the three species, which included visceral organ (e.g. heart, liver and kidney) pallor and hepatic and renal ecchymoses (Figure 5a–e). However, splenic swelling and intestinal haemorrhage was present only among coho salmon and rainbow trout (Figure 5b,c,f; Table 2). Surviving Atlantic salmon, coho salmon and rainbow trout did not exhibit gross external or internal BCWD signs.

Overall, survival ranged from 6.7% in coho salmon to 50.0% to 53.3% in Atlantic salmon and rainbow trout, respectively (Figure 3b). Mortality began 3 days post‐exposure in all species, and peaked on days 6, 7 and 8 in Atlantic salmon, rainbow trout and coho salmon, respectively (Figure 3b). The Cox proportional hazards regression model indicated fish species significantly affected survival (fish species: Wald χ^2^ = 18.22, df = 2, *p*‐value < .0001); Atlantic salmon and rainbow trout were significantly less likely (*p‐*values < .001) to survive than coho salmon, whereas the risk of death among rainbow trout and Atlantic salmon was not significantly different (*p*‐value = .8129).

#### 3.3.4 *Flavobacterium psychrophilum* isolate US62‐ATS

**FIGURE 6:**
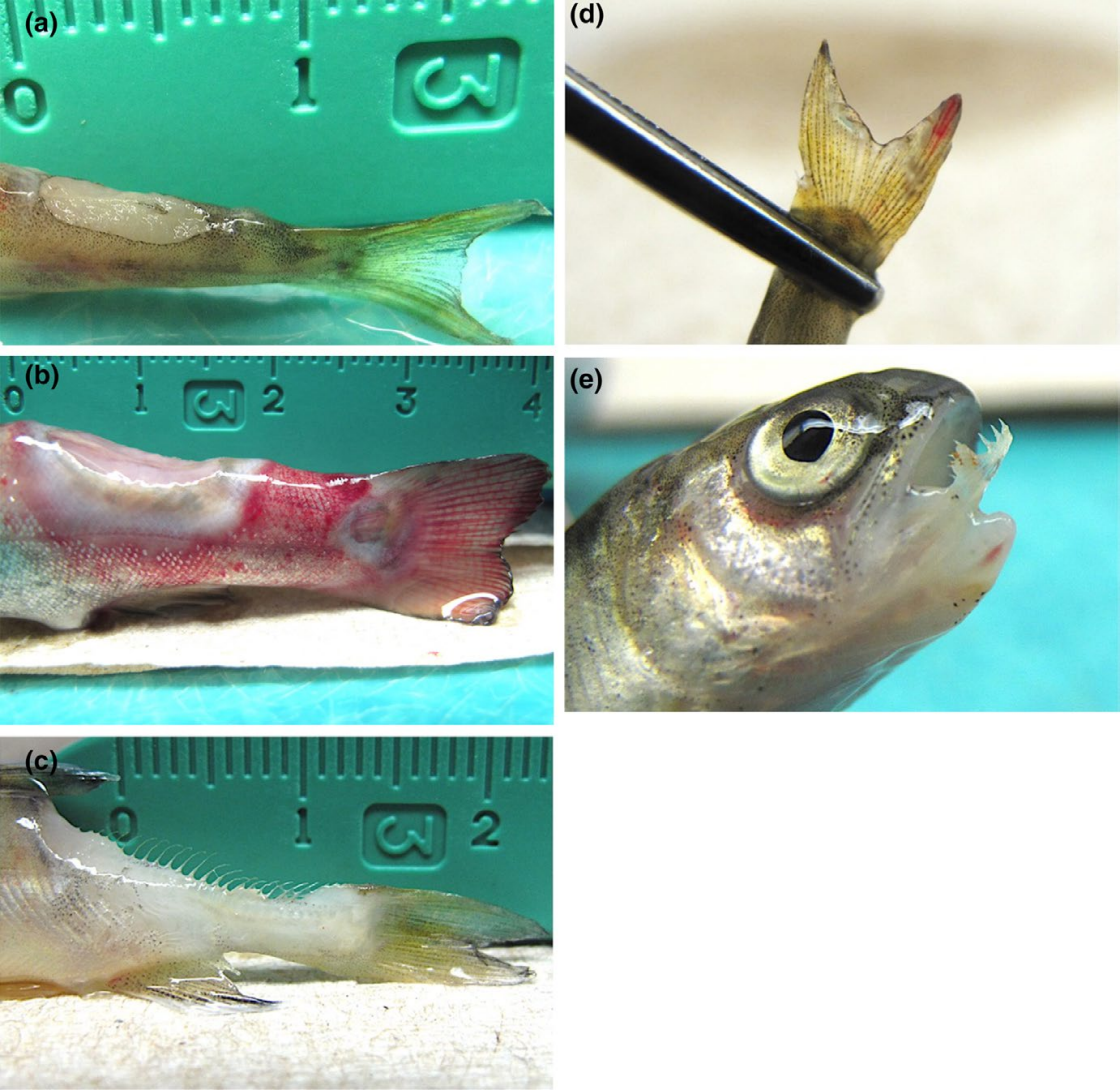
Gross external lesions in Atlantic salmon (*Salmo salar*), coho salmon (*Oncorhynchus kisutch*) and rainbow trout (*O. mykiss*) following immersion challenge with *Flavobacterium psychrophilum* isolate US62‐ATS. (a) Atlantic salmon with focally extensive dermal ulceration of the caudal peduncle that penetrated the underlying musculature. (b) Rainbow trout with multiple, focally extensive dermal ulcerations of the caudal peduncle that penetrated the underlying musculature. Ulceration is surrounded by severe diffuse ecchymoses that extends posteriorly into the caudal fin. (c) Coho salmon with focally extensive ulceration of the caudal peduncle that penetrated the underlying musculature. (d) Atlantic salmon with focal ecchymosis of the caudal fin. (e) Coho salmon with severe peri‐oral ulceration.

Atlantic salmon, rainbow trout and coho salmon exposed to US62‐ATS (ST277, CC‐ST232) developed gross BCWD signs within 2, 3 or 4 days, respectively. All species exhibited focally extensive dermal ulceration of the caudal peduncle (Figure 6a–c). Like rainbow trout exposed to US19‐COS, rainbow trout ulcers were often accompanied by surrounding and severe diffuse ecchymotic haemorrhage that extended posteriorly into the caudal fin (Figure 6b; Table 2). Atlantic salmon also had caudal fin ecchymoses (Figure 6d). In contrast, gross haemorrhage of the caudal peduncle/fin was not present in any coho salmon. Other external BCWD signs common to all fish species included gill pallor with or without ecchymoses and petechiae. A disease sign unique to coho salmon was severe peri‐oral ulceration (Figure 6e). Internal BCWD signs caused by US62‐ATS were identical among all species and included visceral organ (e.g. heart, liver and kidney) pallor and mild splenic swelling (Table 2). Some surviving Atlantic salmon, coho salmon and rainbow trout showed evidence of a prior caudal peduncle ulceration, evidenced by incomplete or complete healing. Internally, surviving fish of all species appeared grossly normal.

Overall survival ranged from 3.3% to 13.3% in Atlantic salmon and rainbow trout, respectively, to 66.7% in coho salmon (Figure 3c). Mortality began 1 (Atlantic salmon), 3 (rainbow trout) and 6 (coho salmon) day(s) post‐exposure and peaked on days 4, 8 and 10 in Atlantic salmon, rainbow trout and coho salmon, respectively (Figure 3c). The Cox proportional hazards regression model indicated fish species significantly affected survival (fish species: Wald χ^2^ = 63.19, df = 2, *p*‐value < .0001), whereby Atlantic salmon and rainbow trout were significantly less likely (*p‐*values < .0001) to survive US62‐ATS infection when compared to coho salmon. Similarly, Atlantic salmon were significantly less likely to survive compared to rainbow trout (*p*‐value < .0001).

### 3.4 Infection status in Atlantic salmon, coho salmon and rainbow trout following immersion exposure to *Flavobacterium psychrophilum* isolates US19‐COS, US62‐ATS and US87‐RBT

#### 3.4.1 Negative control Atlantic salmon, coho salmon and rainbow trout

No bacteria were recovered from any negative control fish throughout these experiments.

#### 3.4.2 *Flavobacterium psychrophilum* isolate US87‐RBT

*Flavobacterium psychrophilum* isolate US87‐RBT was recovered in a pure form and as perfuse lawns (i.e. colony forming units, cfus, too numerous to count) from the caudal peduncle and kidney of all dead rainbow trout (*n =* 13). In surviving rainbow trout, pure cultures of US87‐RBT were recovered from ~35.3% (*n* = 6/17) of the kidney cultures at intensities ranging from 10^0^–10^1^ cfu/g (as determined by calibrated inoculating loops and colony counts) of tissue. Molecular analyses confirmed the recovered bacteria were *F. psychrophilum* and belonged to ST275 (data not shown).

#### 3.4.3 *Flavobacterium psychrophilum* isolate US19‐COS

*Flavobacterium psychrophilum* isolate US19‐COS was recovered in a pure form and as perfuse lawns from the caudal peduncle and kidney of all dead Atlantic salmon (*n =* 15), coho salmon (*n =* 28) and rainbow trout (*n =* 14). *F. psychrophilum* isolate US19‐COS was not recovered from the external or internal tissues of any surviving fish. Molecular analyses confirmed the recovered bacteria were *F. psychrophilum* and belonged to ST13 (data not shown).

#### 3.4.4 *Flavobacterium psychrophilum* isolate US62‐ATS

*Flavobacterium psychrophilum* isolate US62‐ATS was recovered in a pure form and as perfuse lawns from the caudal peduncle ulcerations of all dead Atlantic salmon (*n =* 29) and rainbow trout (*n =* 26). Similarly, US62‐ATS was recovered at intensities ranging from 10^3^ cfu/g to perfuse lawns from the kidney of all dead rainbow trout and most (e.g. *n* = 23/29, 79%) dead Atlantic salmon and in a pure form. In contrast, pure cultures of US62‐ATS were obtained from the caudal peduncle ulcer of 40% (*n* = 4/10) of dead coho salmon at intensities ranging from 10^3^ cfu/g to perfuse lawns. Internally, US62‐ATS was recovered in a pure form from the kidney of all dead coho salmon (*n* = 10), with intensities ranging from 10^3^ cfu/g to perfuse lawns. *F. psychrophilum* isolate US62‐ATS was not recovered from external or internal tissues of any surviving fish. Molecular analyses confirmed the recovered bacteria were *F. psychrophilum* and belonged to ST277 (data not shown).

## 4 DISCUSSION

Herein, results provide evidence that some *F. psychrophilum* MLST variants are host specific, a matter that may affect the development of targeted BCWD prevention and control strategies. Indeed, US87‐RBT (ST275, in CC‐ST10) showed strong infection and disease‐fidelity to rainbow trout, as evidenced by causing disease and subsequent mortality only in rainbow trout. Knupp, Kiupel, et al. (2021) also suggested at least one other CC‐ST10 variant (e.g. ST78) was rainbow trout‐specific after proving avirulent to coho salmon following immersion challenge; however, this study did not also assess the virulence of this variant in rainbow trout. Likewise, Fredriksen et al. (2016) reported that a *F. psychrophilum* isolate highly virulent to rainbow trout was avirulent to Atlantic salmon via injection; however, the MLST genotype of this isolate was not reported. The in vivo findings from this study support the MLST‐based observations that CC‐ST10 appears to be rainbow trout‐specific (Knupp et al., 2019; Nicolas et al., 2008; Nilsen et al., 2014; Van Vliet et al., 2016). In fact, of the 851 *F. psychrophilum* isolates recovered from fish and belonging to CC‐ST10 (https://pubmlst.org/fpsychrophilum), >95% were recovered from *O. mykiss*. Interestingly, the CC‐ST10 isolates that were recovered from other fish species (e.g. white sturgeon, *Acipenser transmontanus*; chinook salmon, *O. tshawytscha*; brown trout, *S. trutta*; coho salmon, Atlantic salmon and *Salvelinus* sp.) may be the result of tightly interconnected fish farming practices. For example, most fish farms in Chile simultaneously rear Atlantic salmon, coho salmon and rainbow trout (Avendano‐Herrera et al., 2020) and thus may partially explain why some CC‐ST10 isolates were recovered from species other than rainbow trout. In addition to being host specific, US87‐RBT was the only isolate recovered 25 days post infection (i.e. the end of the experiment), possibly suggesting it has evolved to circumvent the rainbow trout immune response.

Like US87‐RBT, US19‐COS (ST13, in CC‐ST9) and US62‐ATS (ST277, in CC‐ST232) also caused the most mortality in their host of origin (e.g. coho salmon and Atlantic salmon, respectively). However, and in contrast to US87‐RBT, these *F. psychrophilum* isolates also proved capable of causing disease and mortality in other salmonids, albeit to a lesser degree. Ekman and Norrgren (2003) noted similar findings, whereby an *F. psychrophilum* isolate (MLST variant unknown) recovered from Atlantic salmon caused the most mortality in Atlantic salmon but also caused mortality in rainbow trout and sea trout (*S. trutta* L.). Likewise, Holt (1987) found that although an *F. psychrophilum* isolate (e.g. SH3‐81, in the same CC as US19‐COS; Van Vliet et al., 2016) recovered from coho salmon caused the most mortality in coho salmon, it also caused mortality in Chinook salmon (*O. tshawytscha*) and rainbow trout. However, and notably, these previous studies were conducted via injection, which bypasses some host defences (Dash et al., 2018; Fast et al., 2002) and thereby complicates assessment of the host specificity of the tested variants. Nevertheless, findings herein show that some *F. psychrophilum* variants may have a broader host range, which could have substantial implications for fish farms and hatcheries rearing multiple salmonid species. For instance, these facilities may be at greater risk for widespread losses in the event of a BCWD outbreak in comparison to an outbreak caused by a host specific variant (e.g. ST275). In this context, future studies assessing the transmission dynamics of these *F. psychrophilum* variants and salmonid species are warranted. Beyond the immediate risks, these findings may also affect the development of vaccines intended to protect multiple salmonid species.

Although the primary focus of this study was not to extensively examine the mechanisms underlying *F. psychrophilum* host specificity, observations suggest *O*‐polysaccharide antigenic determinants may play a role. In this context, US87‐RBT belonged to Type‐2 (i.e. serotype Th; Lorenzen & Olesen, 1997; Rochat et al., 2017) and was strongly host specific to rainbow trout. Knupp, Kiupel, et al. (2021) suggested a Type‐2 *F. psychrophilum* variant (e.g. ST78) was rainbow trout specific after proving avirulent to coho salmon via immersion. Indeed, many Type‐2/Th *F. psychrophilum* isolates are virulent to rainbow trout and/or recovered from this species (Avendano‐Herrera et al., 2020; Lorenzen & Olesen, 1997; Sundell et al., 2019). US19‐COS belonged to Type‐0 (i.e. serotype Fp^T^; Knupp, Kiupel, et al., 2021; Lorenzen & Olesen, 1997; Rochat et al., 2017) and caused the most mortality in its host of origin (e.g. coho salmon). These findings are consistent with previous studies, whereby most Type‐0/Fp^T^ *F. psychrophilum* isolates are recovered from coho salmon (Lorenzen & Olesen, 1997; Rochat et al., 2017). Notably, the *F. psychrophilum* type strain (NCIMB 1947^T^) also belongs to Type‐0/Fp^T^ (Lorenzen & Olesen, 1997; Rochat et al., 2017) and is considered avirulent to rainbow trout (Jarau et al., 2018; Madsen & Dalsgaard, 2000; Sundell et al., 2019); however, and given our findings, it seems NCIMB 1947^T^ may not be well‐suited to infect rainbow trout. US62‐ATS belonged to Type‐1 (i.e. serotype Fd; Lorenzen & Olesen, 1997), which contrasts with most previous studies showing Atlantic salmon isolates most often belong to Type‐2 or Type‐4 (Avendano‐Herrera et al., 2020; Rochat et al., 2017). Indeed, most *F. psychrophilum* isolates belonging to Type‐1/Fd are recovered from rainbow trout (Avendano‐Herrera et al., 2020; Lorenzen & Olesen, 1997; Rochat et al., 2017; Saticioglu et al., 2018), and many are virulent to this species via injection (Sundell et al., 2019). Thus, our finding that US62‐ATS belonged to Type‐1 may partially explain the virulence of this isolate to not only Atlantic salmon but rainbow trout. Cisar et al. (2019) reported that serogroup, rather than serotype, more accurately defines Fd, Th and Fp^T^, and thus also applies to the molecular serotypes. Collectively, previous findings and observations herein suggest additional studies characterizing the serotypes of *F. psychrophilum* are needed, a matter that could impact BCWD vaccine development and selective breeding programs.

Another mechanism potentially contributing to *F. psychrophilum* host specificity is proteolytic activity. Rainbow trout‐specific isolate US87‐RBT was the only tested isolate to degrade elastin. Indeed, elastinolytic activity is common among isolates recovered from rainbow trout and belonging to CC‐ST10 (Sundell et al., 2019). However, not all rainbow trout‐recovered *F. psychrophilum* isolates possess this capability (Dalsgaard & Madsen, 2000; Rochat et al., 2019; Soule et al., 2005; Sundell & Wiklund, 2015). Thus, elastinolytic activity may only provide an advantage for some rainbow trout‐associated isolates. The observation that US19‐COS lacks elastinolytic activity is unsurprising given most *F. psychrophilum* isolates recovered from coho salmon and/or belonging to MLST CC‐ST9 and/or serotype Fp^T^ lack this ability (Dalsgaard & Madsen, 2000; Rochat et al., 2019; Soule et al., 2005). Thus, other virulence determinants and/or proteases are sufficient for causing mortality in coho salmon. Indeed, Barbier et al. (2020) reported *F. psychrophilum* isolate OSU THCO2‐90, which was recovered from diseased coho salmon and belongs to CC‐ST9 (i.e. same CC as US19‐COS; Nicolas et al., 2008), secretes at least 49 proteins, including multiple undescribed proteases. Studies assessing the elastinolytic activity of Atlantic salmon‐recovered *F. psychrophilum* isolates have produced mixed results to date (Rochat et al., 2019; Soule et al., 2005; Sundell & Wiklund, 2015). Findings herein clearly show some Atlantic salmon‐recovered *F. psychrophilum* isolates can cause high mortality without this ability. Whether the lack of this trait is common to most Atlantic salmon‐associated *F. psychrophilum* isolates remains to be determined.

This study emphasized the potential role of pathogen specificity. However, disease outcomes result from complex interactions between the pathogen, its host, and its environment (Casadevall & Pirofski, 1999), and previous studies have shown host genetics also play a role in BCWD resistance. For example, Leeds et al. (2010) demonstrated via laboratory challenges that selective breeding was effective at increasing BCWD resistance among rainbow trout. Moreover, multiple quantitative trait loci associated with BCWD resistance in rainbow trout have been identified (Palti et al., 2015; Vallejo et al., 2014; Wiens et al., 2013). Host genetics may contribute to BCWD resistance via differences in immune response. For example, Lee et al. (2023) found that in comparison to a BCWD‐susceptible rainbow trout line, a BCWD‐resistant rainbow trout line had increased expression of M2 macrophages involved anti‐inflammatory responses and tissue repair, and two Toll‐like receptors responsible for pathogen detection and inflammatory response. Nagai and Nakai (2011) found ayu‐recovered *F. psychrophilum* isolates could survive and grow in ayu serum, whereas isolates recovered from salmonids and cyprinids could not. Herein, rainbow trout was the only species with severe diffuse ecchymotic haemorrhage surrounding the caudal peduncle and exophthalmia. Whether the observed differences in diseases signs and mortality among species following exposure to US19‐COS, US62‐ATS and US87‐RBT are due to pathogen and/or host‐derived factors remains to be determined.

In conclusion, we confirmed the MLST‐based observations that some *F. psychrophilum* variants are host specific, whereas others appear more generalistic. We posit the mechanisms driving these disparities are multifaceted, potentially influenced by not only *F. psychrophilum* serotype and secreted proteases but also host genetics and corresponding immune response. The implications of these findings are broad and may affect *F. psychrophilum* transmission dynamics, and the development of effective BCWD vaccines and BCWD‐resistant salmonid lines. Thus, future studies evaluating *F. psychrophilum* host specificity, transmission and the underlying mechanisms are warranted.

## AUTHOR CONTRIBUTIONS

**Christopher Knupp:** Conceptualization; methodology; software; formal analysis; validation; investigation; resources; data curation; writing – original draft; writing – review and editing; visualization; project administration. **Thomas P. Loch:** Conceptualization; methodology; validation; investigation; resources; data curation; writing – original draft; writing – review and editing; funding acquisition; project administration; supervision.

## CONFLICT OF INTEREST STATEMENT

All authors declare that they have no conflict of interest.

## Data Availability

The data that support the findings of this study are available from the corresponding author upon reasonable request.
